# Forward single‐cell sequencing into clinical application: Understanding of ageing and rejuvenation from clinical observation to single‐cell solution

**DOI:** 10.1002/ctm2.827

**Published:** 2022-05-20

**Authors:** Furong Yan, Zhangping Li, Charles A Powell, Xiangdong Wang

**Affiliations:** ^1^ Department of Pulmonary and Critical Care Medicine Zhongshan Hospital, Fudan University Shanghai Medical College Shanghai China; ^2^ Shanghai Institute of Clinical Bioinformatics, Shanghai Engineering Research for AI Technology for Cardiopulmonary Diseases Shanghai China; ^3^ The Quzhou Affiliated Hospital of Wenzhou Medical University, Quzhou People's Hospital Quzhou China; ^4^ Division of Pulmonary Critical Care and Sleep Medicine, Icahn School of Medicine at Mount Sinai New York New York USA

Ageing is characterized by physiological characteristics as the part of natural lifespan and by pathological healthspan processes in response to stress, environmental changes, and diseases. Ageing is a pattern of morphological and functional decline of cells in tissues and circulation, that is correlated with the genetic backgrounds of individuals and with molecular regulatory pathways that may resist ageing influences. The discovery and development of interventions for molecular and cellular rejuvenation is a new and revolutionary field of clinical and translational medicine. Understanding the genetic and epigenetic mechanisms by which health span and lifespan can be regulated, manipulated, and extended is important for identifying and validating “rejuvenating interventions”. These mechanisms include signal regulation, metabolic interference, reprogramming, heterochronic parabiosis, target‐based drugability, and senescent editing.^1^, ^2^, ^3^ One of Clinical and Translational Medicine's missions is to advance spatiotemporal molecular medicine by advancing single‐cell sequencing into clinical application, translating single‐cell measurements into routine practice, and identifying disease‐specific biomarkers and therapies.^4^, ^5^, ^6^ Advanced technologies such as single‐cell RNA sequencing (scRNA‐seq) and spatial transcriptomics have broad applications in clinical tissue or fluid samples to understand intra‐ and intercellular communications and network interactions within the tissue or within the circulation.^7^, ^8^, ^9^, ^10^ The current Editorial extends our understanding of ageing and rejuvenation from clinical observation in clinical and translational medicine to molecular mechanisms of heterochronic parabiosis in the single‐cell solution (Figure 1). The Editorial calls special attention to the identification and development of ageing‐associated and specific biomarkers and targets that may impact ageing metabolism, molecular regulation, and genetic alteration.

**FIGURE 1:**
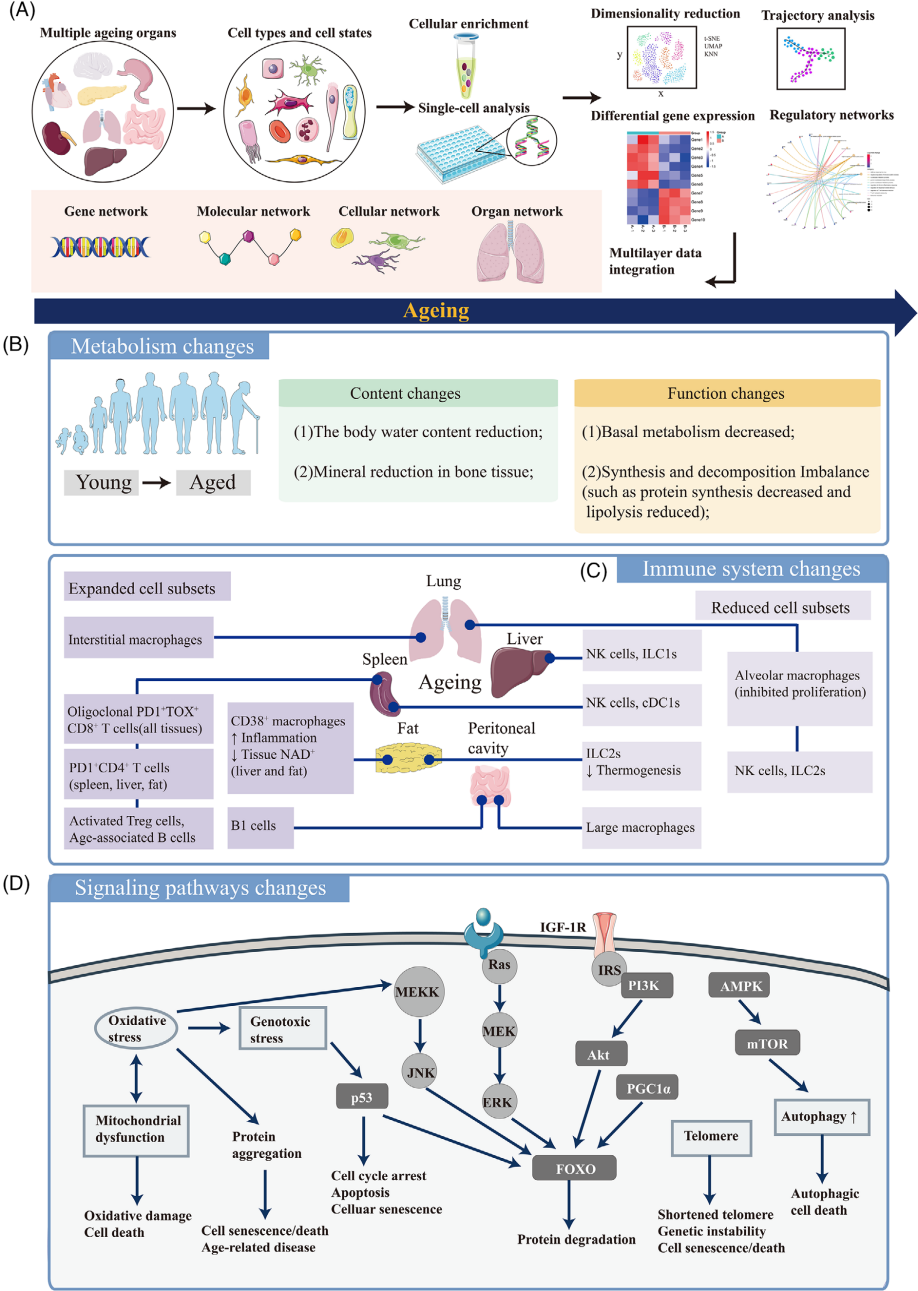
Single‐cell omics in ageing. Ageing is caused by interconnected molecular changes in a wide variety of cells in multiple organs. Single‐cell technology provides a map of human ageing which integrates multiple networks at different biological levels and suggests potential avenues for therapeutic intervention (A). Single‐cell technology can help identify multiple changes in ageing, including the metabolism (B), immune system (C) and signalling pathway changes (D). NK cells: natural killer cells; cDC1: conventional type 1 dendritic cell; ICAM1: intercellular adhesion molecule 1; ILC1s: group 1 innate lymphoid cells; ILC2s: group 2 innate lymphoid cells; PGC1α: peroxisome proliferator‐activated receptor‐γ coactivator‐1α; JNK: c‐Jun N‐terminal kinase; MEKK: mitogen‐activated protein kinase kinase kinase; ERK: extracellular regulated protein kinase; FOXO: Forkhead box O; IGF‐1R: type 1 insulin‐like growth factor receptor; AMPK: AMP‐activated protein kinase; mTOR, mammalian target of rapamycin; PI3K: phosphatidylinositol 3 kinase

Potential targets for influencing ageing are systemic and local metabolic disorders that occur gradually during the ageing process. For example, Melatonin is a functionally pleiotropic indoleamine molecule and a key regulator of energy metabolism that interferes with cell proliferation and growth and induces apoptosis by regulating carboxylesterase 1 expression and epigenetic modification, lipid droplet accumulation, endoplasmic reticulum stress, de novo androgen synthesis.^11^ Xie et al. examined the role of melatonin in ageing‐associated osteoporosis, a common phenome in the elderly.^12^ Using bone marrow stromal cells RNA‐seq, chromatin immunoprecipitation sequencing, and transposase‐accessible chromatin with high‐throughput sequencing, it was shown that downregulation of the histone methyltransferase nuclear receptor binding SET domain protein 2 expression was accompanied by low levels of melatonin in the human bone marrow and was associated with the severity of senile osteoporosis. The sequencing data indicate that the manipulations of molecular metabolisms and epigenetic regulations of melatonin may be a complementary therapy for ageing‐associated diseases. The tripeptide glutathione is a metabolic element that plays important role in balancing intracellular mitochondria‐dominated oxidation, energy production, glucose metabolism, and cell biological behaviours. Preclinical evidence indicates that the deficiency of tripeptide glutathione may accelerate the ageing process from youth to the elderly. Recent 36‐week open‐label clinical trials of supplemental combination of glycine and N‐acetylcysteine in young and older adults demonstrated that the supplementation for 24 weeks could improve the levels of red‐blood‐cell tripeptide glutathione, oxidative stress, mitochondrial function, inflammatory responses, endothelial barrier function, insulin resistance, genomic‐damage, cognition, strength, gait‐speed, exercise capacity, and body‐fat and waist‐circumference.^13^ This is a critical step to translating the concept of metabolic manipulation in anti‐ageing and rejuvenation into the recovery from pathological processes or drug toxicity, although this particular study was still preliminary and has the limitation of a small sample size.

It is a challenge to discover and develop ageing specific biomarkers in the circulation on basis of the metabolites from ageing cells as well as to validate and translate those measurable molecules for clinical practice implementation. Contents of ω‐3 polyunsaturated fatty acids in the human retina play decisive roles in the maintenance of retinal structure and function, and metabolism, and potentially prevent age‐related macular degeneration. Because it is not possible to directly measure retinal ω‐3 content, Acar et al. in the Biomarkers of Lipid Status and Metabolism in Retinal ageing project (2021) assessed lipidomic profiles in the circulation and developed a prediction model to evaluate the predictive strength for determining retinal ω‐3 content.^14^ Of polyunsaturated fatty acids, cholesteryl esters species with ω‐3, rather than those with ω‐6, were correlated with retinal contents. This observation requires further validation in a large population of patients to examine the association between blindness and response to therapy. Zhu et al. investigated trans‐omic profiles of clinical phenomes and lipidomes of patients with lung cancer among lung cancer subtypes, genders, ages, stages, metastatic status, nutritional status, and clinical phenome severity, and found that patterns of circulating lipidomic profiles varied by age.^15^ For example, plasma levels of lysophosphatidylcholine were elevated in patients with lung cancer at ages < 60, lysophosphatidylinositol at ages between 60 and 70, and lysophosphatidylethanolamine at ages > 70. The isomers of the same lipid element can be different across ages of patients with lung cancer, for example, isomers of lysophosphatidylserine 15:1 and 20:3 elevated in ages < 60, while 15:0 and 20:1‐2 in ages between 60–70. Of multiple factors, ageing can change the balance between the electrolyte and water levels in lifespan.^16^

The development of natural molecules‐based interference and lifestyle‐associated reprogramming are considered potential approaches to prevent and treat ageing‐associated dysfunction and diseases. Cell senescence is an important process of cell growth arrest that is dependent upon cellular replication with telomere attrition or stress degrees and is associated with the development of ageing‐related susceptibility and diseases. Of potential therapeutic approaches, the ginsenoside Rg3 (Rg3) was found to have anti‐senescent activity by targeting the calcium release‐activated calcium modulator 1 (ORAI1) protein and activating the adenosine 5‘‐monophosphate (AMP)‐activated protein kinase to promote Ca^2+^ influx into the cytoplasm to induce autophagy and NRF2 activation.^17^ This particular study set an example for exploring the potential for small molecules extracted from natural materials to regulate cell senescence, although the mechanisms of such small molecules are more complex than expected. Rg3 has two epimers 20(S)‐ginsenoside Rg3 (SRg3) and 20(R)‐ginsenoside Rg3 (RRg3) and is one of the ginsenoside family of chemicals extracted from Panax ginseng that has multiple biological interference effects, including anti‐cancer, anti‐angiogenesis, and anti‐inflammation.^18^ In addition, the strategy of a 5‐day water‐only fast was found to alter metabolic‐syndrome and age‐related risk markers and improve immune function, for example, body weight, waist circumference, body mass index, blood pressure, and levels of insulin, insulin‐like growth factor 1, as well as numbers of CD^4+^and CD^8+^ T cells and B cells.^19^ Such strategies could stimulate the recruitment of Treg cells with the potential to increase the systemic capacity of anti‐inflammation and they indicate a new hypothesis that the strategy of living style pattern modifications can be a new alternative for anti‐ageing and rejuvenation.

With rapid development, scRNA‐seq has been widely applied for detecting intercellular heterogeneity and communication, clustering new cell populations in cancer, inflammation, chronic diseases, and ageing. Pálovics et al. investigated inter‐organ and inter‐cellular heterogeneity of transcriptomic profiles in response to heterochronic parabiosis, an approach to exchange blood from young to old or vice versa, and provided solid evidence and new insights for understanding systemic responses and molecular networks among blood‐borne factors and cells at the single‐cell solution.^20^ Deep understanding of heterochronic parabiosis can characterize biological functions and the therapeutic potential of blood‐borne circulating factors in the body, multiple organs, and organelle interactions of ageing. scRNA‐seq data of 20 organs demonstrated that responses to young and aged blood are cell‐type‐specific in heterochronic parabiosis, where adipose mesenchymal stromal cells, haematopoietic stem cells and hepatocytes were most responsive.^20^ scRNA‐seq showed that transcriptomic alterations of skeletal stem cells in ageing were accompanied by the transformation of the bone marrow niche and functional decline.^21^ Such ageing cells can initiate the development of haematopoietic ageing and exhaustion, which can be improved by the combination of bone morphogenetic protein 2 and Colony‐stimulating factor 1, rather than heterochronic parabiosis of young blood or systemic reconstitution with young haematopoietic stem cells. By combining scRNA‐seq, mass cytometry and scATAC‐seq, COVID‐19 was found to promote ageing characteristics of circulating immune cells, for example, T cell polarization from naive and memory cells to effector, cytotoxic, exhausted and regulatory cells, the elevation of late natural killer cells, as well as age‐associated B cells, inflammatory monocytes and dendritic cells.^22^ More work is required to understand the potential of multi‐omics and trans‐omics of single cells to address multiple dimensional understanding of molecular mechanisms on ageing, and translation for clinical and translational medicine remains unclear.

In conclusion, understanding ageing and rejuvenating interventions are one of critical processes of clinical and translational medicine. Understanding the complexity of molecular metabolisms, immune responses, and genetic regulations will be important for the discovery and development of ageing‐associated biomarkers and therapeutic targets. Transcriptomic patterns of circulating immune cells, red blood cells, and platelets at single‐cell resolution can be an important window to examining molecular mechanisms of ageing initiation and development. To translate scRNA‐seq to clinical application, a deep understanding of ageing and rejuvenation from clinical observation to the single‐cell solution will provide opportunities for the innovation and development of new therapeutic strategies.

## CONFLICT OF INTEREST

The authors declare that they have no competing interests.
